# 1-D and 2-D Direction of Arrival Estimation in a Conical Conformal Array: Design and Implementation

**DOI:** 10.3390/s23094536

**Published:** 2023-05-06

**Authors:** Hongyun Zhang, Ping Li, Guangwei Zhang, Guolin Li, Xiaofeng Gao

**Affiliations:** 1 Science and Technology on Electromechanical Dynamic Control Laboratory, School of Mechatronical Engineering, Beijing Institute of Technology, Beijing 100081, China; zhanghongyun@bit.edu.cn (H.Z.); liping85@bit.edu.cn (P.L.); 7920161015@bit.edu.cn (G.L.); 2 Beijing Institute of Radio Measurement, Beijing 100039, China; nmbtgxf@bit.edu.cn

**Keywords:** DOA estimation, conical conformal array, propagator method, manifold separation technique (MST), difference coarray

## Abstract

Direction of arrival (DOA) estimation for conformal arrays is challenging due to non-omnidirectional element patterns and shadow effects. Conical conformal array (CCA) can avoid the shadow effect at small elevation angles. So CCA is suitable for DOA estimation on both azimuth and elevation angles at small elevation angles. However, the element pattern in CCA cannot be obtained by conventional directional element coordinate transformation. Its local element pattern also has connection with the cone angle. The paper establishes the CCA radiation pattern in local coordinate system using 2-D coordinate transformation. In addition, in the case of large elevation angle, only half elements of the CCA can receive signal due to the shadow effect. The array degrees of freedom (DOF) are reduced by halves. We introduce the difference coarray method, which increases the DOF. Moreover, we propose a more accurate propagator method for 2-D cases. This method constructs a new propagation matrix and reduces the estimation error. In addition, this method reduces computational complexity by using linear computations instead of eigenvalue decomposition (EVD) and avoids spectral search. Simulation and experiment verify the estimation performance of the CCA. Both demonstrate the CCA model established in this paper is corresponding to the designed CCA antenna, and the proposed algorithms meet the needs of CCA angle detection. When the number of array elements is 12, the estimation accuracy is about 5 degrees.

## 1. Introduction

DOA estimation is a critical problem in many applications, such as radar, sonar, and wireless communication systems. Conformal arrays, which are composed of sensors placed on a curved surface, have recently received significant attention due to their good aerodynamic performance [1]. Common conformal array shapes are cylindrical conformal arrays [2], conical conformal arrays [3,4], and spherical conformal arrays [5]. These circular array based conformal arrays are the basic structure of most aircraft and are widely used. However, conformal arrays suffer from directional element pattern [6], shadow effect [2], and mutual coupling [7,8,9,10]. Array manifold construction is complicated by these effects, which will corrupt the performance of DOA estimation algorithms. Therefore, current DOA estimation methods cannot be used on CCAs straightforwardly.

To overcome these limitations, several modified DOA estimation algorithms for conformal arrays are suggested. For directive elements, ref. [11] decomposes the radiation pattern by Fourier series and proposes an effective pattern model. Ref. [12] proposed the UCA-RARE algorithm which can successively perform 2-D angle estimation. Ref. [13] optimizes the directivity of the radiation pattern by using CRB. It proved that using directional elements with proper directionality gives better performance than isotropic elements. In order to solve the shadowing effect problem in conformal arrays, ref. [2] proposed the subarray segmentation method, which divides the cylindrical conformal array into several subarrays. For each subarray, the non-isotropic sector array is transformed into a virtual uniform linear array via interpolation method. In order to compensate for the decrease in the array DOF and aperture caused by the shadow effect, ref. [14,15] convert cylindrical conformal arrays to virtual nested arrays [16]. The accuracy of these interpolation algorithms depends on the size of interpolation step, which may introduce additional errors.

Uniform circular arrays (UCAs) are the basis for solving conformal arrays with circular carriers. The phase mode method [17] synthesizes the circular array elements into beamspace composed of several beams. Thus, the steering vector is converted into a product of Bessel function and the exponent of azimuth. The manifold separation technique [18] uses the wavefield modeling [19] to equate the received signal to spatial signal sampling by arbitrary array. The array received signal with arbitrary structure are broken down to the sampling matrix and the Vandermonde structure vector containing angle information. In recent years, corray methods have received extensive attention [16,20,21]. An array contains *N* element can provide $O(N^{2})$ coarray elements by vectorizing the covariance matrix of the received signal. Namely, the array DOF is expanded to $O(N^{2})$. Ref. [22] uses UCA to estimate quasi stationary signals larger than the number of elements by K-R product. Ref. [23] extend the difference coarray concept to arbitrary arrays and perform azimuth estimation of underdetermined signal sources.

Conical conformal arrays can estimate the elevation angle without suffering from shadow effect when the elevation angle is smaller than the cone angle. When the elevation angle is larger than the cone angle, only half of the elements will detect the signal, and the array is suitable for 1-D DOA estimation in this case. CCA is different from general directional UCA and cylindrical conformal array. Its array element pattern not only needs the transformation of azimuth angle, but also the transformation of elevation direction. We establish the local coordinate system radiation pattern of the CCA using 2-D coordinate transformation and propose the CCA-MST method. However, due to the shadow effect, the circular array degenerates into a sector array. In order to recover the array DOF from the sector array, we introduce the difference coarray method and propose the CCA-Coarray algorithm. When the elevation angle is smaller than the cone angle, all array elements can receive signals. The CCA can therefore perform 2-D DOA estimation. Previous beamspace methods generally use eigensubspace methods [24,25]. The propagator method (PM) [26,27] circumvents the EVD step by using steering vectors instead of signalsubspaces. However, when the traditional PM is applied to beamspace, its robustness is poor. This is because the beamspace manifold is regulated by Bessel function. In order to develop the detection performance, we restructure the propagator matrix for the CCA-PM algorithm.

The paper is arranged as follows. First in Section 2, we establish the CCA model, including directional element pattern model and CCA shadow effect model. Section 3 introduces the proposed 1-D CCA-MST method and CCA-Coarray method. Section 4 introduces the proposed 2-D CCA-PM algorithm. Then in Section 5, we analyze the computational complexity of each algorithm, and validate the practice of the various algorithms by simulation. In Section 6 we conduct experimental measurements. Section 7 concludes the paper.

## 2. CCA Signal Model

**Figure 1:**
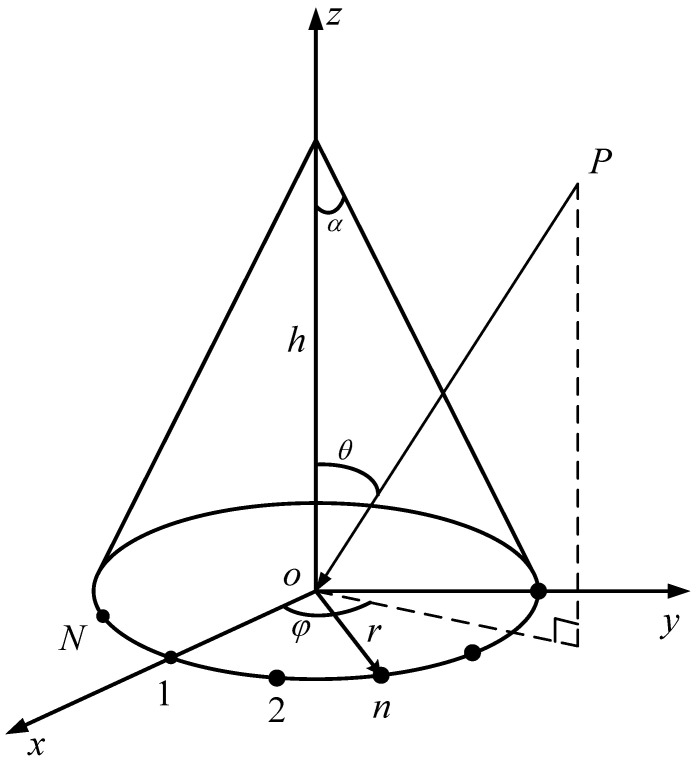
CCA geometry with *N* elements and radius *r*.

The CCA geometry is depicted in Figure 1 with the height of the cone *h* and the cone angle $\alpha \in \left(0,\pi /2\right)$. The base of the cone lies on the xoy plane with the radius *r*. A spherical coordinate system is established with the origin placed at the center of the cone base. The *N* elements are distributed equidistantly over the circumference of the base.The spherical coordinate system is established by taking the center of cone base as the origin. *N* elements are arranged identically along the base circumference.

Assume that *P* narrow band signals with directions $\left(\theta_{p},\varphi_{p}\right),\left(p=1,2,...,P\right)$ incident on the array. The ideal received signal data is then given by

(1) $$X_{e}\left(t\right)=A_{e}s\left(t\right)+n\left(t\right),$$

where $A_{e}=\left[a_{e}\left(\theta_{1},\varphi_{1}\right),a_{e}\left(\theta_{2},\varphi_{2}\right),...,a_{e}\left(\theta_{P},\varphi_{P}\right)\right]$ is the N×P ideal array manifold, s(t) is the P×1 signal matrix and n(t) denote N×1 noise vectors. The ideal steering vector $a_{e}\left(\theta_{p},\varphi_{p}\right)$ are expressed as

(2) $$a_{e}\left(\theta_{p},\varphi_{p}\right)=\left[\begin{array}{c} e^{j\zeta_{p}\cos\left(\varphi_{p}-\phi_{1}\right)}\\ e^{j\zeta_{p}\cos\left(\varphi_{p}-\phi_{2}\right)}\\ \vdots \\ e^{j\zeta_{p}\cos\left(\varphi_{p}-\phi_{N}\right)} \end{array}\right],$$

where $\zeta_{p}=\kappa r\sin\theta_{p},$ κ=2π/λ is the wave number and element angle $\phi_{n}=2\pi \left(n-1\right)/N$, n=1,2,...,N.

**Figure 2:**
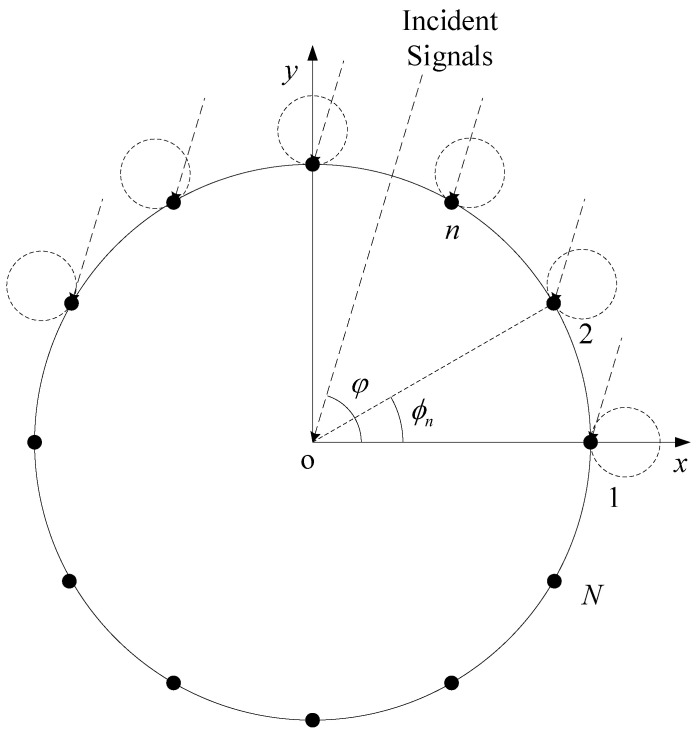
Top view of the CCA. The radiation patterns of different array elements point to different directions.

Each element pattern of the CCA is directional. The antenna element pattern gain is the function of the local coordinate system with elements as the reference point, as shown in Figure 2. For an arbitrary signal, the radiation pattern gains of diverse array elements are different. The directional array elements of circular arrays usually assume that the elevation angle of the maximum directivity of the pattern is $\theta =90^{\circ }$. However, in CCAs, due to the deflection of the elevation angle, the element pattern cannot be obtained by the rotation of the azimuth angle directly. The incident signal angles in the local coordinate system are obtained by the following coordinate transformation:

(3) $$R\left(\phi_{n},\alpha \right)=\left[\begin{array}{ccc} \cos\phi_{n} & -\sin\phi_{n} & 0\\ \sin\phi_{n} & \cos\phi_{n} & 0\\ 0 & 0 & 1 \end{array}\right]\left[\begin{array}{ccc} \cos\left(\frac{\pi }{2}-\alpha \right) & 0 & \sin\left(\frac{\pi }{2}-\alpha \right)\\ 0 & 1 & 0\\ -\sin\left(\frac{\pi }{2}-\alpha \right) & 0 & \cos\left(\frac{\pi }{2}-\alpha \right) \end{array}\right].$$

Assume that all array elements have a 1+cosϕ pattern response, the radiation pattern of the array antenna is represented as

(4) $$\bar{F}\left(\theta ,\varphi -\phi_{n}\right)=1+\cos\left(\theta \right)\cos\left(\frac{\pi }{2}-\alpha \right)+\sin\left(\theta \right)\sin\left(\frac{\pi }{2}-\alpha \right)\cos\left(\varphi -\phi_{n}\right).$$

**Figure 3:**
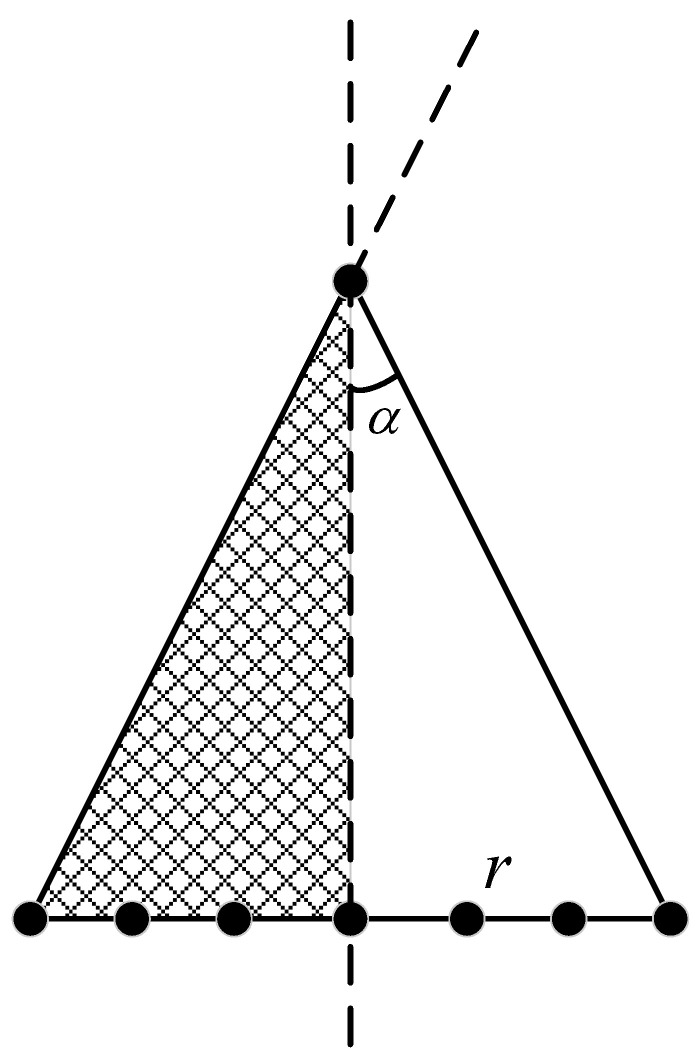
Side view of the CCA. When the elevation angle θ is greater than α, the elements on the left side cannot receive the signal.

The CCA is also affected by the carrier structure, and its received signal has shadow effect. In Figure 3, when the elevation angle θ of a received signal is greater than the cone angle α, only half of the array elements can receive the signal. The rest of the array elements are in the dark side of the carrier. It can be seen from the geometric properties that only when the incident angle in the local coordinate system of the *n*th element satisfies [−π/2,π/2], the *n*th array element can receive the echo signal of the *p*th target. The array that responds to the incident signal is actually a sector array. For analyzing the impact of the shadow effect on CCA target angle estimation, we define the following function *W*

(5) $$W\left(\theta ,\varphi -\phi_{n}\right)=\left\{\begin{array}{c} 1,\;\theta <\alpha \;\mathrm{or}\;\theta >\alpha \;\&\;\left|\varphi -\phi_{n}\right|\leq \frac{\pi }{2}\\ 0,\;\theta >\alpha \;\&\;\frac{\pi }{2}<\left|\varphi -\phi_{n}\right|\leq \pi \end{array}\right..$$

Through the above analysis, the received signal of the CCA is expressed as

(6) $$\begin{array}{cc} X(t) & =W\circ \bar{F}\circ A_{e}s\left(t\right)+n\left(t\right)\\ & =\mathrm{As}(t)+n(t), \end{array}$$

where W is the N×P sign matrix, $\bar{F}$ is the N×P radiation pattern matrix. $A=W\circ \bar{F}\circ A_{e}$ is the N×P CCA manifold matrix, ∘ stands for the Hadamard product. The array response vector (ARV) is given by

(7) $$a\left(\theta ,\varphi \right)=\left[\begin{array}{c} F\left(\theta ,\varphi -\phi_{1}\right)e^{j\zeta \cos(\varphi -\phi_{1})}\\ F\left(\theta ,\varphi -\phi_{2}\right)e^{j\zeta \cos(\varphi -\phi_{2})}\\ \vdots \\ F\left(\theta ,\varphi -\phi_{N}\right)e^{j\zeta \cos(\varphi -\phi_{N})} \end{array}\right],$$

where $F\left(\theta ,\varphi -\phi_{n}\right)=W\left(\theta ,\varphi -\phi_{n}\right)\bar{F}\left(\theta ,\varphi -\phi_{n}\right)$. The covariance matrix is

(8) $$R=E\left[X\left(t\right)X^{H}\left(t\right)\right]=AR_{S}A^{H}+\sigma^{2}I,$$

where $R_{S}=E\left[S\left(t\right)S^{H}\left(t\right)\right]$ is the signal covariance matrix. $\sigma^{2}$ is the variance of the noise. Due to the limited length of the received data, the actual covariance matrix estimation is

(9) $$\hat{R}=\frac{1}{Q}\sum_{t=1}^{Q}X\left(t\right)X^{H}\left(t\right),$$

where *Q* is the number of snapshots.

## 3. 1-D Methods

### 3.1. Manifold Separation on CCA

The radiation pattern $\bar{F}\left(\theta ,\varphi -\phi_{n}\right)$ is a periodic function with period 2π. Therefore, expand it by Fourier series as follows

(10) $$\bar{F}\left(\theta ,\varphi -\phi_{n}\right)=\sum_{k=-K}^{K}C_{k}\left(\theta \right)e^{jk(\varphi -\phi_{n})},$$

where

(11) $$C_{k}\left(\theta \right)=\sum_{n=1}^{N}\bar{F}\left(\theta ,\varphi -\phi_{n}\right)e^{-jk(\varphi -\phi_{n})}.$$

According to the Jacobi-Anger expansion, we expand the *n*th element of the response vector a(θ,φ) as

(12) $$\begin{array}{cc} a_{n}\left(\theta ,\varphi \right) & =\sum_{k=-K}^{K}C_{k}\left(\theta \right)e^{jk(\varphi -\phi_{n})}\sum_{m=-\infty }^{\infty }j^{m}J_{m}\left(\zeta \right)e^{jm(\varphi -\phi_{n})}\\ & =\sum_{k=-K}^{K}\sum_{m=-\infty }^{\infty }C_{k}\left(\theta \right)j^{m}J_{m}\left(\zeta \right)e^{-j\left(k+m\right)\phi_{n}}e^{j(k+m)\varphi }\\ & =\sum_{k=-K}^{K}\sum_{m=-\infty }^{\infty }G_{n,k+m}e^{j(k+m)\varphi }, \end{array}$$

where $G_{n,k+m}=C_{k}\left(\theta \right)j^{m}J_{m}\left(\zeta \right)e^{-j\left(k+m\right)\phi_{n}}$ and $J_{m}\left(\zeta \right)$ is the Bessel function. Suppose the maximum value of *m* is selected as *M*. Substituting Equation (12) into Equation (6), we derive

(13) X(t)=GDs(t)+n(t),

where G is a N×(2M+1)(2K+1) characteristic matrix, and $D=[d\left(\varphi_{1}\right),\ldots ,d\left(\varphi_{P}\right)]$ is a (2M+1)(2K+1)×P matrix with (2K+1) segment Vandermonde structure, $d\left(\varphi \right)={\left[e^{j(-M-K)\varphi },\ldots ,e^{j(M-K)\varphi },\ldots ,e^{j(-M+K)\varphi },\ldots ,e^{j(M+K)\varphi }\right]}^{T}$. Assume that the elevation angle is $90^{\circ }$, constructing root-MUSIC polynomial

(14) $$f\left(z\right)=z^{(2M+1)(2K+1)-1}p^{T}\left(z^{-1}\right)G^{H}U_{N}U_{N}^{H}Gp(z),$$

where $p\left(z\right)={\left[z^{-M-K},\ldots ,z^{M-K},\ldots ,z^{-M+K},\ldots ,z^{M+K}\right]}^{T}$. The noise subspace $U_{N}$ is computed by EVD of R. In Equation (14), the order of the polynomial is 4×(2M+1), and there are 2×(2M+1) pairs of roots. The the angle estimates are obtained by finding the roots of f(z).

### 3.2. The CCA-Coarray Method

Due to the shadow effect, only half of the elements can receive the signal when the elevation angle is large. The DOF of the array also dropped by half. In order to expand the number of incident signals that can be estimated, we propose the CCA-Coarray algorithm in this section.

#### 3.2.1. The Coarray of CCA

Vectorizing R in Equation (8), we obtain

(15) $$z=\mathrm{vec}\left(R\right)=\left(A^{*}⊙A\right)p+\sigma_{n}^{2}1_{n},$$

where $p={\left[\sigma_{1}^{2},\sigma_{2}^{2},\ldots ,\sigma_{P}^{2}\right]}^{T}$ is the signal power vector, $1_{n}=\mathrm{vec}\left(I_{N}\right)$. ⊙ denotes the Khatri–Rao product, $\left(A^{*}⊙A\right)=\left[\bar{a}\left(\theta_{1},\phi_{1}\right),\cdots ,\bar{a}\left(\theta_{P},\phi_{P}\right)\right]$ is the coarray manifold whose sensor positions are given by

(16) $$D=\left\{n_{i}-n_{j},\forall i,j=1,2,\ldots ,N\right\}.$$

The subscripts *i* and *j* represent the corresponding elements, respectively. The vector z is regarded as the received data of a new virtual array with single snapshot, whose array manifold is $A^{*}⊙A$.

The co-ARV is represented as

(17) $$\bar{a}\left(\theta ,\varphi \right)=a^{*}\left(\theta ,\varphi \right)⊗a\left(\theta ,\varphi \right),$$

where ⊗ is the Kronecker product. The *g*th entry of the complete co-ARV is

(18) $$\begin{array}{cc} {\bar{a}}_{g}\left(\theta ,\varphi \right) & =\bar{F}\left(\theta ,\varphi -\phi_{i}\right)\bar{F}\left(\theta ,\varphi -\phi_{j}\right)e^{j\zeta \left(\cos\left(\varphi -\phi_{i}\right)-\cos\left(\varphi -\phi_{j}\right)\right)}\\ & =f_{g}^{\prime }e^{j2\zeta \sin\left(\frac{\phi_{i}-\phi_{j}}{2}\right)\sin\left(\varphi -\frac{\phi_{i}+\phi_{j}}{2}\right)}\\ & =f_{g}^{\prime }e^{j2\zeta \sin\gamma \sin\left(\varphi -\eta \right)}, \end{array}$$

where $\gamma =(\phi_{i}-\phi_{j})/2$, $\eta =(\phi_{i}+\phi_{j})/2$. $f^{\prime }$ is derived in the Appendix A. Since the double angle term in $f^{\prime }$ will cause angular ambiguity, ignoring it we get

(19) $$f_{g}^{\prime }=c_{1}+c_{2}e^{j\varphi }+c_{3}e^{-j\varphi },$$

where

(20) $$\begin{array}{cc} & c_{1}=a^{2}+\frac{1}{2}b^{2}\cos\left(2\gamma \right)\\ & c_{2}=ab\cos\gamma e^{-j\eta }\\ & c_{3}=ab\cos\gamma e^{j\eta }. \end{array}$$

When i=j, vectors D in Equation (16) become zero. These *N* redundant vectors need to be removed. Equation (18) shows that the difference coarray of the CCA forms a uniform concentric circular array (UCCA) composed of ⌊N/2⌋ UCAs. When *N* is odd, each UCA has 2N elements. When *N* is even, each UCA has *N* elements. The radius of each UCA is 2rsinγ. These properties are summarized in Table 1.

#### 3.2.2. MST Based ESPRIT Algorithm

By performing manifold separation on the co-ARVs, Equation (18) can be written as

(21) $$\begin{array}{cc} {\bar{a}}_{g}\left(\theta ,\varphi \right) & =f_{g}^{\prime }\sum_{m=-\infty }^{\infty }j^{m}J_{m}\left(2\zeta \sin\gamma \right)e^{jm\left(\varphi -\eta -\frac{\pi }{2}\right)},\\ & =f_{g}^{\prime }\sum_{m=-\infty }^{\infty }{\bar{G}}_{g,m}^{\prime }e^{jm\varphi }, \end{array}$$

where ${\bar{G}}_{g,m}^{\prime }=J_{m}\left(2\zeta \sin\gamma \right)e^{-jm\eta }$ is the (g,m)th entry of ${\bar{G}}^{\prime }$. The N(N−1)×3(2M+1) CCA coarray characteristic matrix $\bar{G}$ can be represented as

(22) $$\bar{G}=\left[diag\left(c_{1}\right){\bar{G}}^{\prime },diag\left(c_{2}\right){\bar{G}}^{\prime },diag\left(c_{3}\right){\bar{G}}^{\prime }\right].$$

Write the co-ARV into matrix form

(23) $$\bar{a}\left(\theta ,\varphi \right)=\bar{G}\left[\begin{array}{c} {\bar{d}}_{m}\left(\varphi \right)\\ {\bar{d}}_{m+1}\left(\varphi \right)\\ {\bar{d}}_{m-1}\left(\varphi \right) \end{array}\right],$$

where

(24) $${\bar{d}}_{m}\left(\varphi \right)={\left[e^{-jM\varphi },\ldots ,1,\ldots ,e^{jM\varphi }\right]}^{T},$$

is the (2M+1)×1 partly Vandermonde wavefield vector.

Combining Equation (15) and Equation (23), we get

(25) $$z=\bar{G}\bar{D}p+\sigma_{n}^{2}1_{n},$$

where

(26) $$\bar{D}=\left[\begin{array}{ccc} {\bar{d}}_{m}\left(\varphi_{1}\right) & \cdots & {\bar{d}}_{m}\left(\varphi_{P}\right)\\ {\bar{d}}_{m+1}\left(\varphi_{1}\right) & \cdots & {\bar{d}}_{m+1}\left(\varphi_{P}\right)\\ {\bar{d}}_{m-1}\left(\varphi_{1}\right) & \cdots & {\bar{d}}_{m-1}\left(\varphi_{P}\right) \end{array}\right].$$

Left multiplication pseudo-inverse of $\bar{G}$ on both sides of Equation (25)

(27) $$\begin{array}{cc} \bar{z} & ={\bar{G}}^{†}z\\ & =\bar{D}p+\sigma_{n}^{2}{\bar{1}}_{n}, \end{array}$$

where † denote the Moore–Penrose inverse. In order to guarantee the existence of the left inverse matrix of $\bar{G}$, it is required that N(N−1)≥3(2M+1). The pure noise entries in the matrix z caused by shadow effect bring about a reduction in the DOF of the linear array $\bar{D}$. Yet the DOF are upgraded by the difference coarray.

Because the covariance matrix of $\bar{z}$ has the rank of 1, conventional DOA estimation algorithms cannot be directly employed. The first 2M+1 rows of $\bar{D}$ are Hermitian symmetric [28]. The following Toeplitz matrix can therefore be formed

(28) $$\bar{R}=\left[\begin{array}{cccc} {\left[\bar{z}\right]}_{M+1} & {\left[\bar{z}\right]}_{M} & \cdots & {\left[\bar{z}\right]}_{1}\\ {\left[\bar{z}\right]}_{M+2} & {\left[\bar{z}\right]}_{M+1} & \cdots & {\left[\bar{z}\right]}_{2}\\ \vdots & \vdots & \ddots & \vdots \\ {\left[\bar{z}\right]}_{2M+1} & {\left[\bar{z}\right]}_{2M} & \cdots & {\left[\bar{z}\right]}_{M+1} \end{array}\right].$$

Applying the ESPRIT algorithm to $\bar{R}$ gives the estimation of azimuth. It is worth mentioning that this method also works for cylindrical conformal arrays. The specific process of the CCA-Coarray algorithm is shown in Algorithm 1.
**Algorithm 1:** CCA-Coarray Algorithm.Calculate the covariance matrix $\hat{R}$ by Equation (9).Vectorize $\hat{R}$ by Equation (15), and remove the *N* redundant entries.Construct the CCA coarray characteristic matrix $\bar{G}$ by Equation (22).Calculate the Moore–Penrose inverse $G^{†}$ and left multiply z to get $\bar{z}$ in Equation (27).Construct $\bar{R}$ by $\bar{z}$ in Equation (28).Calculate EVD of $\bar{R}$ to get the signalsubspace corresponding to the *P* large eigenvalues.Use the least squares (LS) method to obtain the rotation invariant relationship matrix of the two subarrays in the signal subspace. The DOA estimation can therefore be obtained.

## 4. 2-D Methods

### 4.1. 2-D MUSIC Algorithm

The CCA-MUSIC spectrum is given by [24]

(29) $$f_{MUSIC}=\frac{1}{a^{H}\left(\theta ,\varphi \right)U_{N}U_{N}^{H}a\left(\theta ,\varphi \right)}.$$

Operate 2-D search on the spectrum, the *P* spectral peaks are the arrival angle.

### 4.2. CCA-PM Algorithm

#### 4.2.1. Phase Mode Transform

When the elevation angle θ is smaller than α, the values of the function *W* in the array manifold are all 1. Left multiplies ARV by a weight vector with phase mode *l*, which is given by

(30) $$w_{l}^{H}=\left[e^{jl\phi_{1}},e^{jl\phi_{2}},\cdots ,e^{jl\phi_{n}}\right].$$

The normalized manifold of the *l*th phase mode is given by

(31) $$\begin{array}{cc} b_{l}\left(\theta ,\varphi \right) & =w_{l}^{H}a\\ & =\sum_{n=1}^{N}e^{jl\phi_{n}}F\left(\theta ,\varphi -\phi_{n}\right)e^{j\zeta \cos(\varphi -\phi_{n})}\\ & =\sum_{n=1}^{N}\sum_{k=-K}^{K}C_{k}\left(\theta \right)e^{jk(\varphi -\phi_{n})}e^{jl\phi_{n}}e^{j\zeta \cos(\varphi -\phi_{n})}\\ & =\sum_{n=1}^{N}\sum_{k=-K}^{K}C_{k}\left(\theta \right)e^{j\left(k-l\right)\left(\varphi -\phi_{n}\right)}e^{j\zeta \cos(\varphi -\phi_{n})}e^{jl\varphi }. \end{array}$$

Since $w_{l}^{H}$ also has period of 2π, the beamspace can be given by

(32) $$\begin{array}{cc} b_{l}\left(\theta ,\varphi \right) & =\sum_{k=-K}^{K}C_{k}\left(\theta \right)\left(j^{k-l}J_{k-l}\left(\zeta \right)+\sum_{\rho =-\infty ,\rho \neq 0}^{+\infty }j^{k-l+\rho N}J_{k-l+\rho N}\left(\zeta \right)\right)e^{jl\varphi }\\ & \approx \sum_{k=-K}^{K}C_{k}\left(\theta \right)j^{k-l}J_{k-l}\left(\zeta \right)e^{jl\varphi }. \end{array}$$

The Fourier coefficient $C_{k}\left(\theta \right)$ is determined by the integral

(33) $$C_{k}\left(\theta \right)=\frac{1}{2\pi }\int_{0}^{2\pi }F\left(\theta ,\phi_{n}\right)e^{-jk\phi_{n}}d\phi_{n}.$$

If the pattern in Equation (4) is adopted, then K=1.We can derive

(34) $$\begin{array}{cc} & C_{0}\left(\theta \right)=1+\cos\left(\theta \right)*\cos\left(\frac{\pi }{2}-\alpha \right)\\ & C_{-1}\left(\theta \right)=C_{1}\left(\theta \right)=\frac{1}{2}\sin\left(\theta \right)\sin\left(\frac{\pi }{2}-\alpha \right). \end{array}$$

Substituting Equation (34) into Equation (32), we obtain

(35) $$b_{l}={\bar{b}}_{l}e^{jl\left(\varphi +\frac{\pi }{2}\right)},$$

where the polynomial ${\bar{b}}_{l}=\left(-C_{1}\left(\theta \right)jJ_{l-1}\left(\zeta \right)+C_{0}\left(\theta \right)J_{l}\left(\zeta \right)+C_{1}\left(\theta \right)jJ_{l+1}\left(\zeta \right)\right)$.

When the element pattern is omnidirectional, only the second item $C_{0}$ is included in ${\bar{b}}_{l}$. The first and third items introduced by the directional pattern lead to estimate error. Consider

(36) $$\begin{array}{cc} & {\bar{b}}_{l-1}+{\bar{b}}_{l+1}\\ = & -C_{1}\left(\theta \right)j\left(J_{l-2}\left(\zeta \right)+J_{l}\left(\zeta \right)\right)\\ & +C_{0}\left(\theta \right)\left(J_{l-1}\left(\zeta \right)+J_{l+1}\left(\zeta \right)\right)\\ & +C_{1}\left(\theta \right)j\left(J_{l}\left(\zeta \right)+J_{l+2}\left(\zeta \right)\right). \end{array}$$

Using the following recurrence relation [29]

(37) $$\frac{2l}{\zeta }J_{l}\left(\zeta \right)=J_{l-1}\left(\zeta \right)+J_{l+1}\left(\zeta \right),$$

we derive

(38) $$\begin{array}{cc} \sigma_{b}= & \frac{\zeta }{2}\left({\bar{b}}_{l-1}+{\bar{b}}_{l+1}\right)\\ = & -C_{1}\left(\theta \right)jJ_{l-1}\left(\zeta \right)\left(l-1\right)+C_{0}\left(\theta \right)J_{l}\left(\zeta \right)l+C_{1}\left(\theta \right)jJ_{l+1}\left(\zeta \right)\left(l+1\right)\\ = & l{\bar{b}}_{l}+\left(C_{1}\left(\theta \right)jJ_{l-1}\left(\zeta \right)+C_{1}\left(\theta \right)jJ_{l+1}\left(\zeta \right)\right)\\ = & l{\bar{b}}_{l}+C_{1}\left(\theta \right)j\frac{2l}{\zeta }J_{l}\left(\zeta \right). \end{array}$$

The second term in the last row of Equation (38) is the unwanted term which caused by the directional radiation pattern. The amplitude error of the unwanted term can be ignored and the phase error has the following conclusion.

**Property** **1.** *The phase error between $l{\bar{b}}_{l}$ and $\sigma_{b}$ is*

(39) $$\Delta \approx \arctan\frac{\sin\left(\frac{\pi }{2}-\alpha \right)}{\kappa r\left(1+\cos\left(\frac{\pi }{2}-\alpha \right)\right)}$$

**Proof.**  See Appendix B. □

From Equation (32) we can get the array manifold in beamspace steering vector

(40) $$B=\left(\begin{array}{ccc} b_{1}\left(\theta_{1},\varphi_{1}\right) & \ldots & b_{1}\left(\theta_{P},\varphi_{P}\right)\\ \vdots & b_{l}\left(\theta_{p},\varphi_{p}\right) & \vdots \\ b_{2L+1}\left(\theta_{1},\varphi_{1}\right) & \cdots & b_{2L+1}\left(\theta_{P},\varphi_{P}\right) \end{array}\right).$$

#### 4.2.2. An Improved PM Algorithm

The MUSIC algorithm in Section 4.1 executes EVD and 2-D joint search, which requires huge amounts of computation. The PM circumvents the EVD step by using beamspace manifold instead of signalsubspaces, which cuts down the complexity.

Define the beamspace covariance matrix

(41) $${\hat{R}}_{b}=w_{l}^{H}\hat{R}w_{l}.$$

The recent PM commonly chooses the first *P* rows when blocking the array manifold. However, in CCA beamspace, from Equation (32) we aware that the beamspace manifold is regulated by Bessel function. Low mode numbers correspond to large Bessel function amplitudes. Here we propose a novel block method. Partition the matrix ${\hat{R}}_{b}$ as

(42) $${\hat{R}}_{b}=\left[\begin{array}{ccc} R_{1} & R_{2} & R_{3} \end{array}\right],$$

$\left[\begin{array}{cc} R_{1} & R_{3} \end{array}\right]$ can be obtained by linear transformation of $R_{2}$. That is,

(43) $$\left[\begin{array}{cc} R_{1} & R_{3} \end{array}\right]=R_{2}P,$$

where $P\in C^{P\times (2L+1-P)}$ is the propagator matrix. P is derived by the LS method

(44) $$P=R_{2}^{†}\left[\begin{array}{cc} R_{1} & R_{3} \end{array}\right].$$

Define

(45) $$Q=\left[\begin{array}{c} P_{1}^{H}\\ I_{P}\\ P_{2}^{H} \end{array}\right],$$

where $P_{1}$ is the first $\left\lfloor (2L+1-P)/2\right\rfloor$ column of P, and $P_{2}$ is the last $\left\lceil (2L+1-P)/2\right\rceil$ column of P. Combine Equation (43) with Equation (45) obtains

(46) $$Q{R_{2}}^{H}={\hat{R}}_{b}.$$

Q and B span the same beamspace.

Let $B_{1}$ stand for B with front $L_{2}$ rows, $B_{2}$ stand for B with mid $L_{2}$ rows, $B_{3}$ stand for B with back $L_{2}$ rows, where $L_{2}=2L-1$. Using the recurrence relation in Equation (37) we get

(47) $$\Gamma B_{2}=B_{1}\Phi +B_{3}\Phi^{*},$$

where $\Phi =diag(\sin\theta_{1}e^{j\varphi_{1}},\sin\theta_{2}e^{j\varphi_{2}},\cdots ,\sin\theta_{P}e^{j\varphi_{P}})$ and $\Gamma =\left(2/\kappa r\right)e^{j(\Delta -\pi /2)}diag\left\{-\left(L-1\right),\cdots ,0,\cdots ,\left(L-1\right)\right\}$. Δ is given by Equation (39). Let $Q_{1}$ stand for Q with front $L_{2}$ rows, $Q_{2}$ stand for Q with mid $L_{2}$ rows, $Q_{3}$ stand for Q with back $L_{2}$ rows. In a same manner we get

(48) $$\Gamma Q_{2}=Q_{1}\Psi +Q_{3}\Psi^{*},$$

where $\Psi =B_{y}\Phi B_{y}^{-1}$. $B_{y}$ is a nonsingular matrix taken from the middle of B with the largest amplitude. Ψ and Φ hence contain identical eigenvalues $\lambda_{p}=\sin\theta_{p}e^{j\varphi_{p}},p=1,2,\cdots P$. Equation (48) can also be written as

(49) $$\Gamma Q_{2}=\left[\begin{array}{cc} Q_{1} & Q_{3} \end{array}\right]\left[\begin{array}{c} \Psi \\ \Psi^{*} \end{array}\right],$$

namely

(50) $$\left[\begin{array}{c} \Psi \\ \Psi^{*} \end{array}\right]={\left[\begin{array}{cc} Q_{1} & Q_{3} \end{array}\right]}^{†}\Gamma Q_{2}.$$

Calculating EVD of Ψ can obtain the automatically paired angles

(51) $$\begin{array}{c} \theta_{p}=\mathrm{arc}\sin\left(\left|\lambda_{p}\right|\right)\\ \varphi_{p}=∠\lambda_{p} \end{array}.$$

The CCA-PM method is concluded in Algorithm 2.
**Algorithm 2:** The CCA-PM Algorithm.Calculate the beamspace covariance matrix ${\hat{R}}_{b}$ by Equation (41).Calculate the propagator matrix P by Equation (44). Then acquire Q by Equation (45).Partition Q and construct matrix $\left[Q_{1}\;Q_{3}\right]$ as Equation (49).Calculate ${\left[\Psi^{T}\;\Psi^{H}\right]}^{T}$ by Equation (50).Compute the eigenvalues of the front *P* rows of ${\left[\Psi^{T}\;\Psi^{H}\right]}^{T}$. The automatically paired angles are acquired by Equation (51).

## 5. Simulation Results

The Cramer-Rao bound (CRB) of the CCA is given by [30,31]

(52) CRB=σ22NRe(DHΠA⊥D)⊙[1⊗(RsAHR−1ARs)]T−1,

where $\sigma^{2}$ is the noise variance, $1=\left(\begin{array}{cc} 1 & 1\\ 1 & 1 \end{array}\right)$ is a 2 × 2 matrix of 1. $D=[\frac{\partial a_{1}}{\partial \theta_{1}},\frac{\partial a_{2}}{\partial \theta_{2}},\ldots ,\frac{\partial a_{P}}{\partial \theta_{P}},\frac{\partial a_{1}}{\partial \varphi_{1}},\frac{\partial a_{2}}{\partial \varphi_{2}},\ldots \frac{\partial a_{P}}{\partial \varphi_{P}}]$. $\Pi_{A}^{⊥}=I_{2L+1}-A{\left(A^{H}A\right)}^{-1}A^{H}$ is the projection matrix of A. $a_{p}$ is the *p*th column of A.

The RMSE is given by

(53) $$\mathrm{RMSE}=\frac{1}{P}\sum_{p=1}^{P}\sqrt{\frac{1}{500}\sum_{\xi =1}^{500}\left[{\left({\hat{\theta }}_{\xi ,p}-\theta_{p}\right)}^{2}+{\left({\hat{\varphi }}_{\xi ,p}-\varphi_{p}\right)}^{2}\right]},$$

where ${\hat{\theta }}_{\xi ,p}$ and ${\hat{\varphi }}_{\xi ,p}$ represents the estimated values of *p*th angles in the ξth test. $\theta_{p}$ and $\varphi_{p}$ are the real angles of the *p*th incident signal. The CRB and RMSE in the 1-D scenario are special cases of 2-D and will not be repeated here.

### 5.1. Computational Complexity

For the CCA-Coarray method, calculating the covariance matrix $\hat{R}$ requires $N^{2}Q$ flops. The calculation of $G^{†}$ can be processed offline in advance, so it does not need to be considered. Left multiply z by $G^{†}$ requires 3N(N−1)(2M+1) flops. The ESPRIT algorithm requires $M^{3}+2P^{2}M+3P^{3}$ flops. For the CCA-PM algorithm, computing the beamspace covariance matrix ${\hat{R}}_{b}$ needs $\left(2L+1\right)NQ+{\left(2L+1\right)}^{2}Q$ flops. The calculation of the propagator matrix P requires $P{\left(2L+1\right)}^{2}+P^{2}\left(2L+1\right)$ flops. The calculation of the rotation matrix requires $6P^{2}\left(2L-1\right)+12P^{3}$ flops. The specific content is summarized in Table 2.

**Figure 4:**
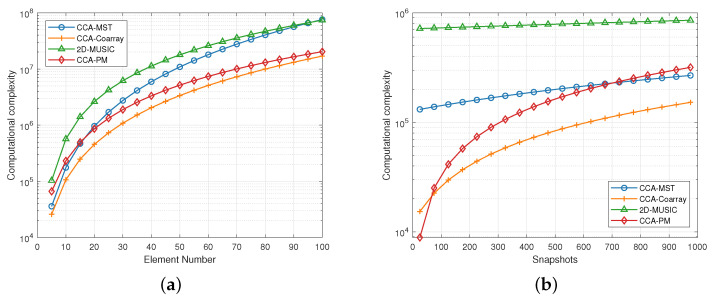
(**a**) Description of the computational complexity contrast of the methods. The element number *N* ranges from 5 to 100 and the snapshots Q=1000. (**b**) The snapshots *Q* ranges from 25 to 1000 and element number N=12.

We use the following parameters for simulation. The signal sources number is *P* = 2. The search points are *T* = 3240. The mode number L=N/2. The expansion number M=N. The computational complexity comparison of varied methods are demonstrated in Figure 4.

It can be seen from Figure 4 that the computational complexity of the CCA-Coarray algorithm is low, followed by the CCA-PM algorithm. However, the CCA-MST algorithm and the 2D-MUSIC algorithm have high computational complexity because they need to perform polynomial root finding and spectral peak search respectively. In 1-D estimations, the coarray-based algorithm can use the inverse matrix of the character matrix G to preprocess the covariance matrix due to the increase in the dimension of the steering vector. The new covariance matrix can use the ESPRIT algorithm. Compared with MUSIC-based algorithms, the complexity is reduced. On the other hand, considering the omnidirectional angle estimation, the interpolation method needs to select multiple sub-arrays for estimation which requires more computations. In 2-D estimation, the proposed PM algorithm avoids element level EVD and spectral searches which reduces computational complexity.

### 5.2. Performance Analysis on 1-D Methods

To validate the estimation performance of the methods, a CCA with *N* = 12, radius r=λ and cone angle $\alpha =33.5^{\circ }$ is adopted. The interpolation step is $0.1^{\circ }$. In each subject, 500 Monte Carlo experiments are conducted. The following simulations consider 2 incident signal at $\varphi_{1}=-150^{\circ },\varphi_{2}=60^{\circ }$.

#### 5.2.1. RMSE

**Figure 5:**
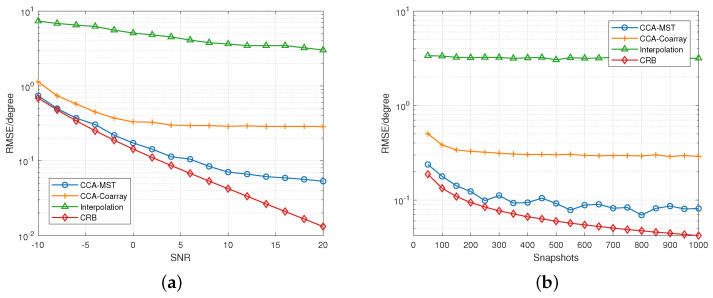
(**a**) RMSE vs. SNR between algorithms. The SNR range is −10 dB to 20 dB and snapshots is 1000. (**b**) RMSE vs. snapshots between the algorithms. The snapshots range is 50 to 1000 and SNR = 10 dB.

Figure 5a demonstrates the RMSE varying with SNR. Figure 5b demonstrates the RMSE varying with snapshots. Figure 5 illustrate RMSE of MST-based algorithms is decreased than conventional interpolation method in conformal arrays. CCA-MST trades high computational complexity for estimation accuracy close to CRB. At 4dB, 1000 snapshots, the estimation error of CCA-Coarray method is less than $0.3^{\circ }$.

#### 5.2.2. DOF Expansion

**Figure 6:**
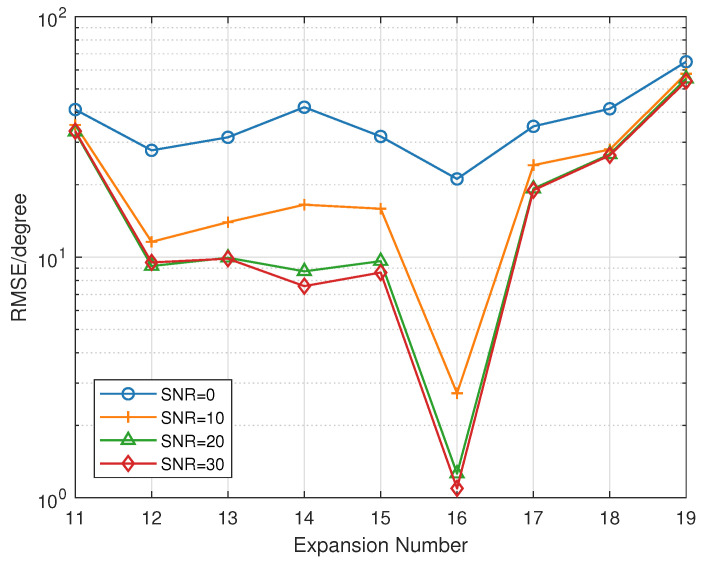
The relationship between between RMSE and and expansion number when snapshots is 1000. The expansion number varies from 11 to 19. Different lines are for different SNR.

The coarray technique can increase antenna aperture and angular DOF. Suppose there are 6 signals incident into the array from $\left[-150^{\circ },150^{\circ }\right]$. The relationship between RMSE and manifold separation expansion number *M* is shown in Figure 6. It can be seen that *M* has a suitable value range. If *M* is too small, the truncation error of Jacobi-Anger expansion is large. When *M* is too large, the Bessel function tends to 0, which will affect the performance of the inverse matrix $G^{†}$. From the simulation results, M=16 seems to be the optimal choice when the number of element number N=12.

### 5.3. Performance Analysis on 2-D Methods

#### 5.3.1. CRB and the Elevation Angle

**Figure 7:**
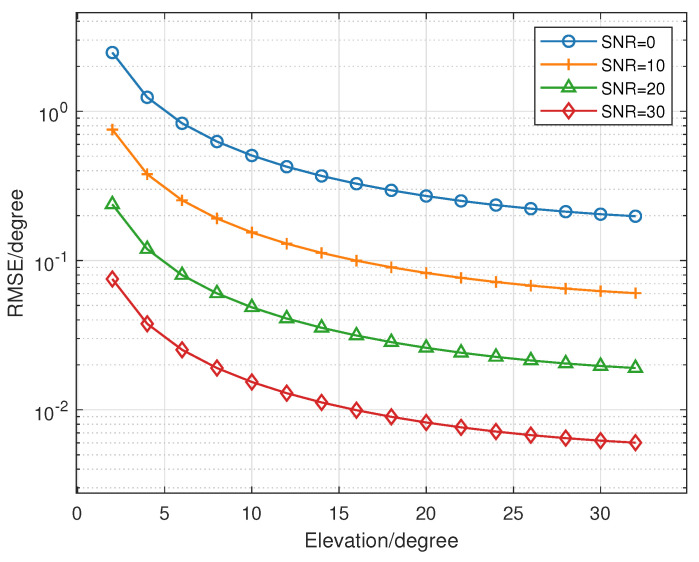
The relationship between the elevation angle and CRB when snapshots is 1000. The elevation angle changes from 0 to 32. The different lines are for different SNR.

Due to the influence of circular array structure and element radiation pattern, the CRB of CCA elevation angle is connected to the ture angle. Figure 7 shows the relationship between elevation angle and CRB. It shows the smaller the elevation angle, the larger the estimation error.

#### 5.3.2. RMSE and Probability of Detection

**Figure 8:**
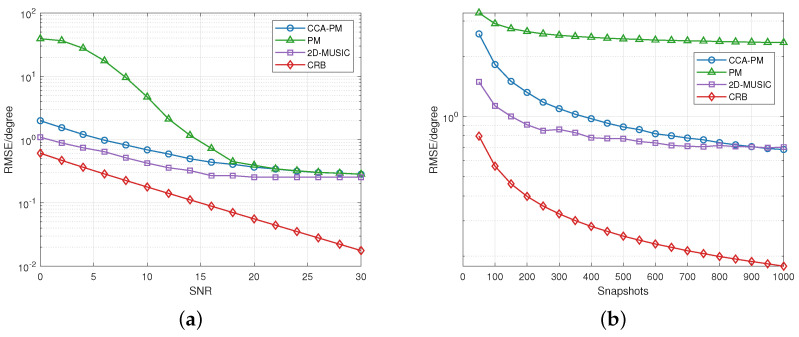
(**a**) RMSE versus SNR between the algorithms. The SNR range is 0 dB to 30 dB and snapshots is 1000. (**b**) RMSE versus snapshots between the algorithms. The snapshots range is 50 to 1000 and SNR = 10 dB.

The following simulations consider 2 different targets locating at ($\theta_{1}=10^{\circ }$, $\varphi_{1}=-110^{\circ }$) and ($\theta_{2}=30^{\circ },\varphi_{2}=45^{\circ }$). Figure 8a demonstrates the RMSE varying with SNR. Figure 8b demonstrates the RMSE varying with snapshots. Figure 8 illustrate RMSE of the proposed PM method is decreased than conventional PM methods. At 6 dB, 1000 snapshots, the estimation error of CCA-PM method is less than $1^{\circ }$.

**Figure 9:**
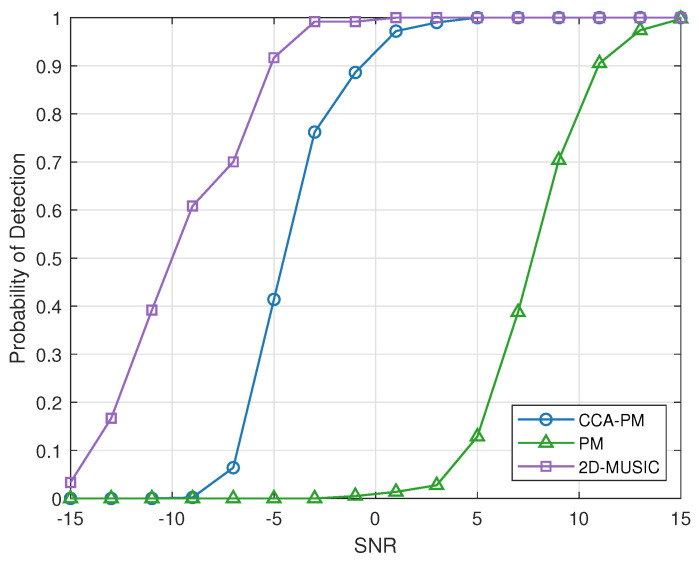
The detection performance versus SNR between the methods. The snapshots is 1000 and SNR varied from −15 dB to 15 dB.

Figure 9 demonstrates the probability of detection of the methods with changing SNR. The purpose is to demonstrate the performance of the proposed algorithms at lower SNR.

## 6. Experimental Results

**Figure 10:**
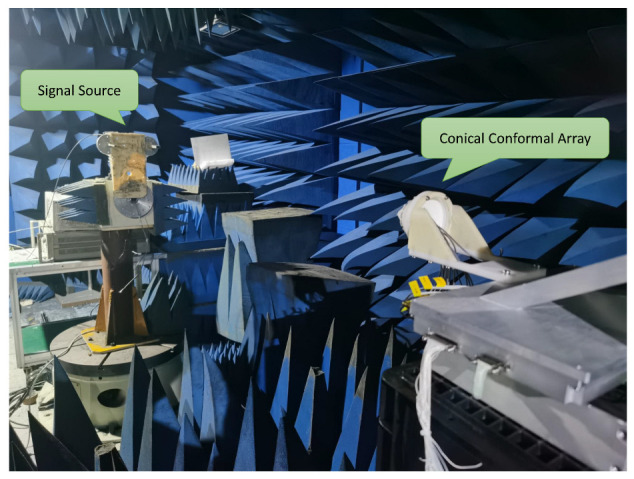
Measurement scene in a microwave anechoic chamber.

**Figure 11:**
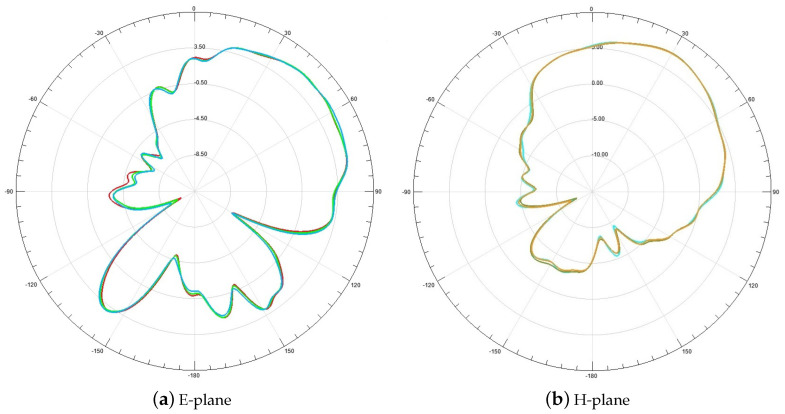
The E-plane (**a**) and H-plane (**b**) of the radiation pattern of an element.

To verify the proposed CCA DOA estimation method, a CCA detection prototype based on LFMCW system is built. The experiment scene is shown in Figure 10. The axis of the cone is horizontal to the ground, and the signal sources are placed at 2.8m from the CCA. The working parameters of CCA are shown in Table 3. Wherein, the E-plane and H-plane of the radiation pattern of the first element is given by Figure 11. After excluding mutual coupling and magnitude-phase consistency errors, the results are shown in Table 4. It can be seen that the experimental results can verify the simulation results. Since only half of the array elements are used for 1-D DOA estimation, its estimation accuracy is low.

## 7. Conclusions

In summary, 1-D and 2-D DOA estimation of a conical conformal array is studied. First, the CCA geometric model and signal model are established to solve the issues of directional pattern and shadow effect. Then, the CCA-MST, CCA-Coarray and CCA-PM algorithms are proposed, which are suitable for 1-D and 2-D scenarios, respectively. The results show that in presence of 2 targets, the estimation error of the CCA-Coarray method is less than $0.3^{\circ }$ at 4 dB with 1000 snapshots. The estimation error of the CCA-PM method is less than $1^{\circ }$ at 6 dB under 1000 snapshots. The method is also more efficient than traditional subspace methods. In the future, the CCA-based mutual coupling compensation algorithm remains to be explored.

## Figures and Tables

**Table 1:** Properties of the Virtual UCCA.

	Number of UCAs	Elements per UCA	All Difference Sensors
**Even**	N/2	*N*	$N^{2}/2$
**Odd**	(N−1)/2	2N	$N^{2}-N$

**Table 2:** Computational complexity of diverse methods.

	Method	Computational Complexity
1-D Methods	CCA-MST	$N^{2}Q+N^{3}+3N\left(2M+1\right)+\left(N+1\right)\left(N-P\right)+{\left(4\left(2M+1\right)\right)}^{3}$
	CCA-Coarray	$N^{2}Q+3N\left(N-1\right)\left(2M+1\right)+M^{3}+2P^{2}M+3P^{3}$
	Interpolation	$2P(\frac{1}{2}N^{2}Q+\frac{1}{8}N^{3}+P^{2}N+3P^{3})$
2-D Methods	2-D MUSIC	$N^{2}Q+N^{3}+T\left(N\left(N-P\right)+{\left(N-P\right)}^{2}\right)$
	CCA-PM	$\left(2L+1\right)NQ+{\left(2L+1\right)}^{2}Q+P{\left(2L+1\right)}^{2}+14P^{2}L+13P^{3}$
	CCA-ESPRIT	$\left(2L+1\right)NQ+{\left(2L+1\right)}^{2}Q+{\left(2L+1\right)}^{3}+6P^{2}\left(2L-1\right)+13P^{3}$

**Table 3:** CCA design parameters.

Parameter	Value
Carrier Frequency $f_{c}$	12 GHz
Wavelength λ	2.5 cm
Antenna Gain	5 dB
VSWR	Average 1.24
Radiation Efficiency	68%
Element Number *N*	12
Radius of the Array *r*	2.26 cm
Cone Angle α	$33.5^{\circ }$
High *h*	3.4 cm
Snapshots *Q*	1024

**Table 4:** Measurement results.

	(a) CCA-MST	
	Target 1	Target 2
Target Angle	$\theta =15^{\circ },\varphi =0^{\circ }$	$\theta =15^{\circ },\varphi =60^{\circ }$
Estimated Value	$\varphi =4.5^{\circ }$	$\varphi =65.5^{\circ }$
Error	$4.5^{\circ }$	$5.5^{\circ }$
	**(b) CCA-Coarray**	
	Target 1	Target 2
Target Angle	$\theta =15^{\circ },\varphi =0^{\circ }$	$\theta =15^{\circ },\varphi =60^{\circ }$
Estimated Value	$\varphi =7.5^{\circ }$	$\varphi =69^{\circ }$
Error	$7.5^{\circ }$	$9^{\circ }$
	**(c) CCA-PM**	
	Target 1	Target 2
Target Angle	$\theta =15^{\circ },\varphi =0^{\circ }$	$\theta =15^{\circ },\varphi =60^{\circ }$
Estimated Value	$\theta =13.3^{\circ },\varphi =4.2^{\circ }$	$\theta =13.5^{\circ },\varphi =65.1^{\circ }$
Error	$4.5^{\circ }$	$5.3^{\circ }$

## Data Availability

The code can be requested from the author by e-mail.

## Appendix A

Let

(A1) $$\begin{array}{cc} & a=1+\cos\left(\theta \right)*\cos\left(\frac{\pi }{2}-\alpha \right)\\ & b=\sin\left(\theta \right)\sin\left(\frac{\pi }{2}-\alpha \right). \end{array}$$

The coarray pattern is

(A2) $$\begin{array}{cc} f^{\prime } & =\bar{F}\left(\theta ,\varphi -\phi_{i}\right)\bar{F}\left(\theta ,\varphi -\phi_{j}\right)\\ & =\left[a+b\cos\left(\varphi -\phi_{i}\right)\right]\left[a+b\cos\left(\varphi -\phi_{j}\right)\right]\\ & =a^{2}+ab\left[2\cos\left(\varphi -\eta \right)\cos\gamma \right]+\frac{1}{2}b^{2}\left[\cos\left(2\gamma \right)+\cos\left(2\varphi -2\eta \right)\right]\\ & =a^{2}+\frac{1}{2}b^{2}\cos\left(2\gamma \right)+2ab\cos\gamma \left(\frac{e^{j(\varphi -\eta )}+e^{-j(\varphi -\eta )}}{2}\right)+\frac{1}{2}b^{2}\left(\frac{e^{j(2\varphi -2\eta )}+e^{-j(2\varphi -2\eta )}}{2}\right)\\ & =a^{2}+\frac{1}{2}b^{2}\cos\left(2\gamma \right)+ab\cos\gamma e^{-j\eta }e^{j\varphi }+ab\cos\gamma e^{j\eta }e^{-j\varphi }+o\left(\varphi \right). \end{array}$$

## Appendix B

For simplicity, assume x>0. Let

(A3) $$\begin{array}{cc} & x=C_{0}\left(\theta \right)mJ_{m}\left(\zeta \right)\\ & y=C_{1}\left(\theta \right)mJ_{m+1}\left(\zeta \right)-C_{1}\left(\theta \right)mJ_{m-1}\left(\zeta \right)\\ & z=C_{1}\left(\theta \right)\frac{2m}{\zeta }J_{m}\left(\zeta \right). \end{array}$$

So $ℜ\left(m{\bar{b}}_{m}\right)=ℜ\left(\sigma_{b}\right)=x$ and $ℑ(m{\bar{b}}_{m})=y$ and $ℑ(\sigma_{b})=y+z$. The angle error between $m{\bar{b}}_{m}$ and $\sigma_{b}$ is

(A4) $$\begin{array}{cc} \Delta & =\arctan\frac{y+z}{x}-\arctan\frac{y}{x}\\ & =\arctan\frac{xz}{x^{2}+y\left(y+z\right)}. \end{array}$$

Since θ is smaller than α, in the case of a small elevation angle, the cosθ is approximately 1. Since *y* is much smaller than *x*, *y* is ignored here.

(A5) $$\Delta \approx \arctan\frac{z}{x}\approx \arctan\frac{\sin\left(\frac{\pi }{2}-\alpha \right)}{kr\left(1+\cos\left(\frac{\pi }{2}-\alpha \right)\right)}.$$
